# Regulatory effects of protein S-acylation on insulin secretion and insulin action

**DOI:** 10.1098/rsob.210017

**Published:** 2021-03-31

**Authors:** Luke H. Chamberlain, Michael J. Shipston, Gwyn W. Gould

**Affiliations:** ^1^ Strathclyde Institute of Pharmacy and Biomedical Sciences, University of Strathclyde, Glasgow, UK; ^2^ Centre for Discovery Brain Sciences, Edinburgh Medical School: Biomedical Sciences, University of Edinburgh, Edinburgh, UK

**Keywords:** S-acylation, zDHHC enzymes, acyl protein thioesterase, insulin secretion, insulin signalling, GLUT4

## Abstract

Post-translational modifications (PTMs) such as phosphorylation and ubiquitination are well-studied events with a recognized importance in all aspects of cellular function. By contrast, protein S-acylation, although a widespread PTM with important functions in most physiological systems, has received far less attention. Perturbations in S-acylation are linked to various disorders, including intellectual disability, cancer and diabetes, suggesting that this less-studied modification is likely to be of considerable biological importance. As an exemplar, in this review, we focus on the newly emerging links between S-acylation and the hormone insulin. Specifically, we examine how S-acylation regulates key components of the insulin secretion and insulin response pathways. The proteins discussed highlight the diverse array of proteins that are modified by S-acylation, including channels, transporters, receptors and trafficking proteins and also illustrate the diverse effects that S-acylation has on these proteins, from membrane binding and micro-localization to regulation of protein sorting and protein interactions.

## Graphical abstract

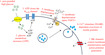

## 1. Introduction

S-acylation, the attachment of fatty acyl chains onto cysteine residues, is a prominent post-translational modification (PTM) affecting a broad and diverse collection of proteins [1–3]. This modification appears on thousands of eukaryotic proteins and regulates many aspects of protein biology, including membrane interaction, intracellular sorting and stability [1–3]. There is no defined consensus motif for S-acylation, but modified cysteines are typically present adjacent to other lipid chains (e.g. *N*-myristoyl or prenyl modifications), within hydrophobic domains or in membrane-proximal regions of transmembrane proteins. Indeed, membrane proximity is a fundamental requirement for cysteine S-acylation as the zDHHC enzymes that mediate this process are all transmembrane proteins with the catalytic site positioned at the membrane bilayer [4,5].

The study of S-acylation has accelerated over the last decade or so, stimulated to a large extent by methodological breakthroughs. Although traditional ^3^H-palmitate labelling is still an important research tool for the field [6], this has been complemented by more recently developed techniques including acyl-biotin exchange (ABE), acyl resin-assisted capture (acyl-RAC) and click chemistry [2,7–11]. In addition to increased sensitivity, these newer techniques also support the purification of S-acylated proteins, which has facilitated the analysis of the S-acylated proteome from a range of different cell and tissue types, and highlighted the diverse nature of S-acylated proteins [12,13].

S-acylation is distinguished from other lipid modifications of proteins, such as *N*-myristoylation, prenylation and glypiation due to its reversibility [13,14]. Dynamic S-acylation is regulated by the opposing activities of acyltransferase and thioesterase enzymes. S-acyltransferases belong to the zDHHC enzyme family and there are 23 distinct *ZDHHC* genes in humans encoding polytopic membrane proteins that contain a conserved 51-amino acid cysteine-rich domain (CRD) within which there is a highly conserved aspartate–histidine–histidine–cysteine (DHHC) motif [15–18]. This DHHC tetrapeptide is the catalytic site of zDHHC enzymes and the cysteine residue reacts with acyl-CoA in the first step of the enzymatic process to form an S-acylated intermediate, which is followed by transfer of the acyl chain to a cysteine in the substrate protein [19,20]; the first step in the reaction is commonly referred to as ‘autoacylation’ and the autoacylation status of zDHHC enzymes is often used as a proxy for enzyme activity. Deacylation is also an enzyme-mediated process, and the acyl protein thioesterases APT1 (encoded by the *LYPLA1* gene) and APT2 (*LYPLA2*) are the best-characterized deacylation enzymes [21], although more recent work has shown that members of the ABHD family also mediate protein deacylation [22,23]. S-acylation turnover rates vary dramatically, with some proteins displaying *t*_1/2_ values in the order of a few minutes and other proteins displaying no detectable turnover during their lifetime, presumably reflecting that they are not recognized by, or accessible to, thioesterase enzymes [13].

The importance of S-acylation is underscored by the fact that this PTM is essential for the functions of most physiological systems, and defects in the process are linked to many human pathologies, including cancer, neurological disorders and diabetes [2]. In this review, we focus on the function of S-acylation within the endocrine system, and specifically on its potential role in insulin secretion from pancreatic β-cells and insulin action in adipocytes and skeletal muscle. Our discussion includes studies on S-acylation in other cell types where it can potentially inform on processes occurring in β-cells and insulin-responsive tissues. The main proteins that are discussed in the review and their key properties are summarized in table 1.

**Table 1.** S-acylated proteins in β-cells and insulin-responsive cells. The table highlights whether the proteins are TM (transmembrane) or soluble, the likely S-acylated cysteines, and the major zDHHC and thioesterase enzymes that are thought to control their S-acylation.

protein name	protein type	modified cysteines	main zDHHC enzyme	thioesterase enzyme
Kir6.2	TM	C166	ND	APT1/2
β2a	soluble	C3, C4	ND	ND
BK α subunit	TM			
* S0–S1*		C53, C56	zDHHC23	APT1, Lyplal1
* STREX*		C645, C646	zDHHC17	ABHD17a/c
GLP-1R	TM	C438	ND	ND
SNAP25	soluble	C85, C88, C90, C92	zDHHC17	ND
Syt-7	TM	C35, C38, C41	ND	ND
ClipR-59	soluble	C534, C535	zDHHC17	ND
SNAP23	soluble	C79, C80, C83, C85, C87	zDHHC17	ND
sortilin	TM	C783	ND	ND
GLUT4	TM	C223	zDHHC7	ND
IRAP	TM	C103, C114	ND	ND

## 2. Insulin secretion

**Figure 1.**
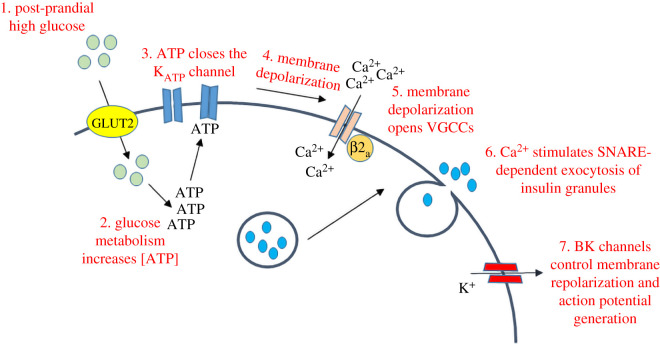
Glucose-stimulated insulin secretion in pancreatic β-cells. The steps in the pathway are indicated in the figure (steps 1–7).

**Figure 2.**
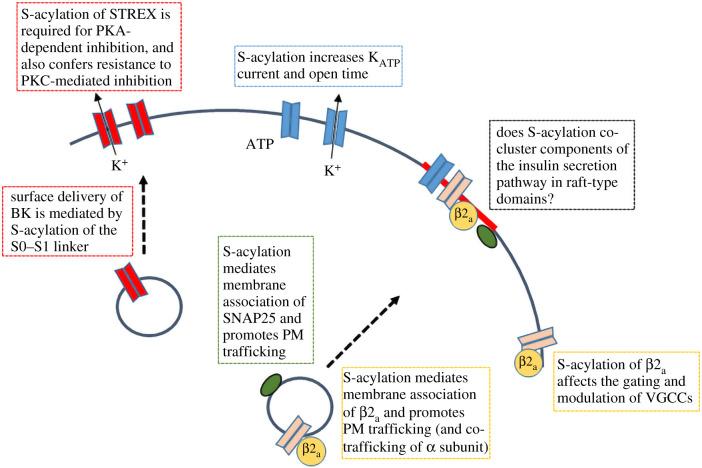
S-acylation of components of the glucose-stimulated insulin secretion pathway. The figure summarizes the key effects of S-acylation on mediating membrane binding, trafficking and channel gating and modulation. S-acylation has also been suggested to mediate the association of SNAP25 with lipid rafts [29,30] and although there is no evidence linking S-acylation and raft association of channels in β-cells, we speculate that S-acylated components of the insulin secretion pathway might co-localize in defined microdomains to enhance the efficiency of signalling. VGCC, voltage-gated Ca^2+^ channel.

Insulin is the most important hormone regulating glucose metabolism in the body. Insulin is produced by β-cells in the pancreas, initially in the form of preproinsulin, which is translocated across the membrane of the endoplasmic reticulum during its biosynthesis [24]. Following cleavage of the signal peptide (pre sequence), proinsulin begins its journey through the secretory pathway and during this process is cleaved to generate insulin (A and B chain joined by disulfide bonds) and C-peptide. Insulin is stored in a Zn^2+^-stabilized hexameric state in secretory granules that form at the Golgi. These granules undergo fusion with the plasma membrane (or ‘exocytosis’) primarily in response to a rise in the level of glucose in the blood [24,25] (summarized in figure 1). Glucose-stimulated insulin secretion occurs as a result of changes in metabolism and ion fluxes in the β-cells. Specifically, increased glucose entry through GLUT2 leads to increased glucose phosphorylation by glucokinase and a consequent rise in the ATP : ADP ratio as a result of enhanced flux through glycolysis and the TCA cycle. This rise in ATP : ADP causes the closure of ATP-gated potassium channels (K_ATP_—see below). Decreased efflux of K^+^ from the β-cells contributes to membrane depolarization and the opening of voltage-gated Ca^2+^ channels, the activity of which is also controlled by other ion fluxes controlling membrane potential. The resulting Ca^2+^ influx stimulates SNARE protein-dependent fusion of insulin granules with the plasma membrane and secretion of insulin from the β-cell [25]. Insulin secretion occurs in two phases: the first phase has been proposed to occur due to the rapid exocytosis of a ‘readily-releasable’ pool of granules, whereas the second-phase insulin secretion may reflect the recruitment of ‘newcomer’ granules to the membrane and their subsequent exocytosis [25]. Several SNARE proteins are involved in insulin granule exocytosis including syntaxin 1 and SNAP25 (plasma membrane SNAREs), and vesicle-associated membrane protein-2 (VAMP-2) on the insulin granule membrane [25]. Additional SNAREs appear to function in granule exocytosis that contributes to second-phase insulin secretion, including syntaxin-3/-4 and VAMP8 [25]. Calcium-secretion coupling involves members of the synaptotagmin protein family, in particular synaptotagmin-7 [26] and synaptotagmin-9 [25,27,28]. Many key proteins in insulin biosynthesis/secretion are S-acylated and thus represent potential regulatory sites (figure 2). Below, we explore some examples.

### 2.1. S-acylation and regulation of ion channels controlling β-cell physiology and insulin secretion

S-acylation is a major determinant in the control of a diverse array of ion channels [2]— from assembly through trafficking to the plasma membrane and regulation of channel gating and modulation by intracellular signalling cascades. While the full array of ion channels that are S-acylated in β-cells remains to be determined, channels important for control of β-cell physiology and insulin secretion, including K_ATP_, voltage-gated Ca^2+^ channels and large conductance calcium- and voltage- activated (BK) channels, are potently regulated by S-acylation (figure 2).

#### 2.1.1. S-acylation of Kir6.2 affects K_ATP_ channel activity

Functional K_ATP_ channels consists of four Kir6.x subunits, which assemble together with 4 SUR subunits, and in pancreatic β-cells these channels are composed of Kir6.2 and SUR1 subunits. A recent study using ABE assays on co-transfected HEK293 cells suggested that Kir6.2 (but not SUR1) is modified by S-acylation [31]. Similar experiments performed in rat cardiomyocytes confirmed the S-acylation of Kir6.2 and showed that this modification was enhanced following incubation of the cells with either palmitate or a combination of ML348 and ML349, selective inhibitors of the thioesterases APT1 and APT2, respectively [31]. Electrophysiological recordings of inside-out membrane patches showed that treatment with palmitate or ML348/ML349 promoted an increase in mean patch current and open time and decreased sensitivity to ATP; these effects were seen for endogenous channels in rat cardiomyocytes and INS1 pancreatic β-cells, and in HEK293 cells transfected with Kir6.2 together with SUR1 or SUR2A [31]. Assuming that the effect of these treatments reflects changes in the S-acylation of Kir6.2, it is likely that S-acylation regulates the gating or the modulation of K_ATP_ activity as cell surface levels of the channel were unaffected [31] (figure 2). Indeed, further analysis suggested that palmitate and ML348/ML349 destabilized the closed state of the channel and increased sensitivity to phosphatidylinositol 4,5-bisphosphate (PIP2), which promotes the channel transition from closed to open state [31].

Mutational analysis identified cysteine-166, which sits at the cytosol-membrane interface, as one potential S-acylation site in Kir6.2 [31]. Indeed, a Kir6.2 mutant with a cysteine-166 to valine substitution did not exhibit changes in either S-acylation or activity following cell treatment with palmitate or ML348/ML349 [31]. Although the function and/or localization of transmembrane proteins is often affected by S-acylation, in most cases the mechanisms underlying the regulatory effects of this modification have not been defined. For Kir6.2, structural modelling suggested that the S-acyl chain attached to cysteine-166 on one monomer could form contact with PIP2 that is associated with another Kir6.2 monomer, altering the packing of transmembrane helices and increasing the open state of the channel [31], and thus providing an explanation for the observed functional effects of palmitate and ML348/ML349 on K_ATP_ activity. The proposed effects of S-acylation on cysteine-166 on channel open state probability would be likely to inhibit insulin secretion, which requires K_ATP_ closure to mediate membrane depolarization, and it will be important to test this prediction in future work.

In addition to altering membrane packing of channel helices, S-acylation might exert additional regulatory effects on the K_ATP_ channel. Indeed, it is interesting to note that although the C166 V mutant did not show enhanced S-acylation in response to either palmitate or ML348/ML349, the mutant channel was still S-acylated [31]. This suggests that there may be additional (non-dynamic) S-acylated cysteines in Kir6.2, which might exert other effects, for example, on channel trafficking and micro-localization at the plasma membrane. The modelling analysis performed by Yang *et al.* [31] highlighted the potential interaction of the S-acyl chain attached to cysteine-166 with PIP2 present on a different channel subunit. Similarly, the S-acyl chains (either at cysteine-166 or other sites in the channel) might also mediate specific interactions with membrane lipids, which could alter membrane micro-localization, and this will be interesting to explore in future work. Effects of S-acylation on protein interaction with cholesterol-rich lipid-ordered domains at the plasma membrane have been documented for a number of transmembrane proteins, including the sodium–calcium exchanger NCX1, which displays S-acylation-dependent accumulation in lipid-ordered domains that is linked to transporter function [32].

A central aspect of the analysis of Kir6.2 cysteine-166 S-acylation is its sensitivity to cell treatment with palmitate and ML348/ML349. To understand the importance of S-acylation at this site for β-cell function it will therefore be important to characterize the dynamic nature of cysteine-166 S-acylation and how, for example, it responds to glucose signalling. Similarly, quantitative analysis of K_ATP_ S-acylation will also be important to determine what fraction of the channel is modified at this cysteine under steady-state conditions. Analysis of the presence of S-acylation at other sites in K_ATP_ will also be important to begin to support a comprehensive analysis of the wider effects of S-acylation on K_ATP_ biology. Other key questions include the following. (i) Is APT1/APT2 activity sensitive to glucose signalling or other metabolic changes that occur in β-cells? In this regard, it is interesting to note that APT1 activity was reported to be downregulated in human umbilical vein endothelial cells in hyperglycaemic conditions and also in endothelial cells in db/db mice, which display morbid obesity and chronic hyperglycaemia [33]. Thus, chronic hyperglycaemic conditions might mimic ML348/ML349 treatment by blocking APT1 function, thereby promoting enhanced S-acylation of K_ATP_ and leading to defects in insulin secretion. (ii) Are channel S-acylation kinetics affected in type 2 diabetes, either through changes in APT1/APT2 (see above) or via changes in zDHHC enzyme activity? Here, it is also interesting to note that specific substrate recruitment to zDHHC5 is regulated by GlcNAcylation of this enzyme [34], which is of interest given that this PTM has been linked hyperglycaemia [35]. (iii) Does this modification affect sensitivity to sulfonylureas? Indeed, it is interesting to note that mutations at cysteine-166 are associated with neonatal diabetes further highlighting the potential clinical relevance of K_ATP_ S-acylation [36].

#### 2.1.2. S-acylation affects the trafficking, activity and modulation of voltage-gated Ca^2+^ channels

Modulation of K_ATP_ activity by ATP leads to membrane depolarization and the opening of voltage-gated Ca^2+^ channels on the β-cell membrane, providing a cytosolic Ca^2+^ signal that stimulates insulin granule exocytosis. Different classes of voltage-gated Ca^2+^ channels have been implicated in insulin secretion. In mice, β-cell-specific depletion of the α_1_C pore-forming subunit of Ca_v_1.2 L-type channels had a major effect on glucose-stimulated first-phase insulin secretion *in vivo* [37]. Human β-cells, on the other hand, appear to have a more prominent role of P/Q (Ca_v_2.1) channels, which work in concert with L-type and T-type (Ca_v_3) channels to promote glucose-stimulated insulin secretion [38].

Functional voltage-gated Ca^2+^ channels consist of hetero-oligomeric assemblies of the pore-forming α_1_ subunit together with the auxiliary β, α_2_δ and γ subunits [39]. The β subunits are encoded by four different genes (*CACNB1–CACNB4*) and the β2_a_ subunit is unique in undergoing S-acylation on two N-terminal cysteine residues (cysteine-3 and cysteine-4) [40]. The β2 subunit has been shown to be highly expressed in human islets and two of the most abundant transcripts corresponded to β2_a_ [41]. Comparison of the localization of wild-type and a cysteine-to-serine mutant (C3S/C4S) of β2_a_ in rat INS-1 β-cells highlighted the importance of S-acylation for plasma membrane targeting [41,42]. Furthermore, plasma membrane localization of the Ca_v_1.2 α_1_C subunit was also increased when co-expressed in COS-1 cells with wild-type β2a, compared with the S-acylation-null mutant [41] (figure 2).

In addition to effects on membrane targeting, S-acylation of the β2_a_ subunit has also been reported to impact the activity and modulation of different voltage-gated Ca^2+^ channels (figure 2). In the first study reporting S-acylation of β2_a_, the electrophysiological analysis suggested that mutation of the S-acylation sites led to a reduction in current carried by α_1_C in co-transfected tsA201 cells [40]. This occurred without changes in charge movement, which suggested that similar amounts of the channel were expressed at the plasma membrane [40] (although it is unclear how this relates to the subsequent observation of reduced plasma membrane targeting of the β2_a_ S-acylation mutant [42]). The functional impact of β2_a_ S-acylation was further highlighted by a subsequent study which suggested that *all* electrophysiological features of β2_a_ that distinguish this protein from the other β subunits when expressed in *Xenopus* oocytes are lost when the S-acylation sites are mutated [43]. For example, a cysteine mutant form of β2_a_, in contrast to the wild-type protein, was able to support pre-pulse facilitation of α_1_C currents to a similar level as other β subunits [43].

What are the mechanisms underlying the effects of β2_a_ S-acylation on functional properties of Ca^2+^ channels (beyond membrane targeting)? Some potential insight into this question came from studies investigating the modulation of N-type (Ca_v_2.2) channels by Gq-coupled G protein-coupled receptors (GqPCRs). There are several mechanisms that have been proposed to underpin the slow modulation of voltage-gated Ca^2+^ channels by GqPCR activation, including lipid modulation as a result of reduced PIP2 levels and/or increased levels of arachidonic acid (AA). By using either GqPCR agonists or direct application of AA to HEK-M1 cells, it was shown that the type of modulation (enhancement or inhibition) elicited on N-type channels was dependent on the specific β subunit that was present [44]. For all β subunits other than β2_a_, channels were inhibited, whereas with β2_a_ the current was enhanced [44]. This difference was linked to S-acylation of β2_a_ because mutation of the S-acylation sites switched channel enhancement to channel inhibition [44]. To explain these effects, the authors proposed that AA (released following the actions of PLA_2_) binds to an inhibitory site on the N-type channels and that access to this site is blocked (or occupied) by S-acyl chains on the β2_a_ subunit [44]. In support of this model, deletion of two amino acids in the α_1_B pore-forming subunit of N-type calcium channels that alters the orientation of the α and β subunits with respect to each other led to the S-acylated β2_a_ subunit showing a similar inhibitory effect as the other β subunits [44]. This experiment supports the idea that the exact position of the S-acyl chains in the Ca^2+^ channel complex is important to exert the unique regulatory effects of β2_a_. Another study showed that mutation of the S-acylation sites in β2_a_ increased N-type Ca^2+^ channel inhibition following PIP2 depletion in HEK293 tsA-201 cell line, further showing a link between S-acylation and lipid-mediated, voltage-independent modulation of Ca^2+^ channel activity [45]. These experiments highlight a possible direct role for β2_a_ S-acylation in regulating the activity of voltage-gated Ca^2+^ channels via direct interaction with regulatory sites in the α subunit.

The effects of β2_a_ S-acylation on GqPCR regulation of Cav2.2 were also shown to extend to the voltage-dependent modulation by Gβγ, this time with the β2_a_ subunit-containing channels displaying more pronounced inhibition than channels containing β2_b_, β_3_ or a β2_a_ mutant with substitution of the S-acylated cysteines [46]. In addition to highlighting mechanisms underpinning cross-signalling between GPCRs and Ca^2+^ channels that might be relevant to β-cell function and insulin secretion, these studies are interesting because they highlight the potential of S-acylation of one subunit to modify the function of another subunit within a protein complex via direct interactions with modulatory domains. Indeed, this is reminiscent of the proposal that S-acyl chains on one Kir6.2 subunit can interact with PIP2 bound to another Kir6.2 subunit in the K_ATP_ channel complex (see the section above on K_ATP_). Collectively, the existing data on β2_a_ subunit S-acylation highlight the importance of this modification for trafficking of the subunit to the plasma membrane, co-trafficking of the pore-forming subunit and regulation/modulation of channel activity. Progress in this area requires a detailed assessment of β2_a_ subunit S-acylation dynamics in β-cells and how manipulation of S-acylation affects channel activity in this cell type. As with K_ATP_, it will be interesting to explore the potential effects of S-acylation on membrane micro-localization of the β2_a_ subunit and the Ca^2+^ channel complex. An intriguing possibility is that S-acylation of the K_ATP_ and voltage-gated Ca^2+^ channels promotes functional coupling of these ion channels at the plasma membrane (figure 2).

#### 2.1.3. BK channels are regulated by S-acylation and also play an important role in glucose-stimulated insulin secretion

As glucose-induced insulin release is mediated through changes in the electrical excitability of pancreatic β-cells, mechanisms that control action potential amplitude and repolarization are important determinants of calcium influx and insulin exocytosis. In both human and mouse β-cells, action potential amplitude and repolarization are regulated by large conductance voltage- and calcium-activated (BK) potassium channels [38,47–50]. BK channels are potent determinants in regulating voltage-gated Ca^2+^ influx and exocytosis in many cell types; indeed, acute pharmacological inhibition of BK channels has been reported to increase glucose-stimulated insulin secretion in both human and mouse islets [38,47,48]. It may thus seem surprising that mice with a global genetic deletion of BK channels (BK^−/–^) display reduced, rather than improved, glucose tolerance [51]. Furthermore, although insulin secretion at basal glucose levels was unaffected, at higher glucose concentrations (greater than 10 mmol l^−1^) there was a reduced insulin release in isolated islets from (BK^−/−^) mice [51]. However, genetic loss of BK channels, or long-term pharmacological inhibition of BK channels, also results in significantly reduced β-cell viability and increased susceptibility to oxidative stress and increased apoptosis [51]. Taken together, this may indicate BK channel activity plays a dual role: in the short term (by allowing more Ca^2+^-entry) reduced BK channel activity stimulates insulin secretion [38,47,48]; in the longer term, it leads to β-cell exhaustion and β-cell death [51]. Thus, mechanisms that control either (i) the number of functional BK channels or (ii) the intrinsic BK channel activity or regulation at the plasma membrane are likely to be critical determinants of pancreatic β-cell function.

S-acylation controls the trafficking, properties and regulation of BK channels via multifactorial mechanisms through S-acylation of both the pore-forming α-subunit as well as regulatory subunits [52]. The pore-forming α-subunit of BK channels, encoded by the single gene *KCNMA1*, is S-acylated at two different sites with distinct functional consequences (figure 2). Firstly, a conserved cluster of cysteine residues (Cys 53 and Cys 56 in mouse [53–55]) in the intracellular linker between transmembrane segments S0–S1 can control cell surface delivery of BK channels as well as the interaction with regulatory β-subunits [53–55]. S-acylation of the S0–S1 site by zDHHC23 is required for efficient forward trafficking of the pore-forming subunit alone to the plasma membrane. Indeed, genetic knockdown of zDHHC23, or site-directed mutagenesis of Cys53 : 56 to alanine results in reduced cell surface expression and enhanced retention of the α-subunit in the endoplasmic reticulum [53–55]. The S0–S1 site is de-acylated by the thioesterases APT1 (Lypla1) and the related APT1-like thioesterase (Lyplal1) resulting in enhanced retention in the *trans* Golgi network [55,56]. Lyplal1 has been associated with type 2 diabetes [57] including through use of first-phase insulin secretion as a marker to identify candidate interacting SNPs [58]. BK channels that are de-acylated in the S0–S1 loop also display reduced lateral mobility in the plasma membrane [59] but do not affect the intrinsic calcium- and voltage sensitivity of the channel encoded by the α-subunit alone [54]. However, additional complexity arises as in many tissues, including pancreas, BK channels assemble with regulatory β-subunits and thus the functional outcome of S0–S1 S-acylation in pancreatic β-cells is not known. For example, assembly of the α-subunit with the β1-subunit, that is highly expressed in vascular smooth muscle cells, overrides the deficit in cell surface delivery of the de-acylated α-subunit but reduces the functional coupling between α- and β1-subunit. Thus, while BK channels are efficiently delivered to the cell surface, their activity is reduced as the normal effect of the β1-subunit to shift the voltage for half-maximal activity into the more physiological range of negative potentials is attenuated when the S0–S1 loop is de-acylated [53]. In addition, the regulatory β4 subunit that is expressed in many endocrine tissue including the pancreas is itself S-acylated and the S-acylated β4-subunit can enhance cell surface expression of a-subunits by facilitating ER exit [60]. The role of S-acylation in controlling BK channel properties determined by other regulatory β or γ-subunits is not known. For example, both mouse and human β-cells display BK currents that show rapid inactivation [38,47] that may result from assembly with regulatory β2-subunits [61] and/or one of the recently identified members of the γ-subunit family, LINGO1 [62]. An important question that remains to be resolved is the molecular composition of BK channels in pancreatic β-cells and the extent to which the S0–S1 site and regulatory subunits may be dynamically S-acylated.

Secondly, the α-subunit may also be S-acylated in an alternatively spliced insert encoded by the stress regulated exon (STREX) located in the C-terminus of the channel between the two RCK domains. Based on single-cell qRT-PCR analysis the STREX splice variant appears to be the dominant splice variant expressed in murine pancreatic β-cells [51] and is also expressed in the human pancreas. The BK channel α-subunit that lacks the STREX insert (ZERO variant) is also expressed at lower levels in mouse β-cells; however, the relative contribution of these different variants to β-cell physiology is unknown [51]. An siRNA-based screen revealed the STREX insert is S-acylated by multiple zDHHCs including the plasma membrane localized zDHHC5 and Golgi located zDHHC17, with zDHHC17 being the major zDHHC controlling the STREX domain in HEK293 cells [63,64]. Conversely, STREX is almost exclusively de-acylated by the plasma membrane localized thioesterase ABHD17a (also by ABHD17c but not the related ABHD17b) [56]. Thus, STREX is S-acylated and de-acylated by distinct enzymes compared to those that control S-acylation of the conserved S0–S1 domain. STREX is S-acylated at a dicysteine motif (cysteine-645 and cysteine-646 in mouse) that allows the STREX domain to interact with the plasma membrane. This S-acylation-dependent membrane association is important for the enhanced apparent calcium sensitivity of the STREX splice variant, compared to splice variants that lack the STREX insert [63,64]. Importantly, STREX variant channels are implicated in the control of action potential regulation and electrical bursting behaviour in a number of endocrine cell types, including in the pituitary and adrenal glands [65,66]. S-acylation of STREX has no effect on surface delivery of BK channels but importantly determines the regulation of BK channels by the AGC family of protein kinases [63,67]. S-acylation of the STREX variant of BK channels is required for protein kinase A (PKA) dependent inhibition of the channel (in contrast to ZERO variant channels, which lack STREX and are typically activated by PKA phosphorylation) [63,67]. Conversely, S-acylated STREX variants are resistant to inhibition by protein kinase C (PKC) dependent phosphorylation, whereas deacylation of the STREX domain prevents PKA-mediated inhibition but now allows PKC dependent inhibition [63,67]. Thus, the S-acylation status of STREX acts as a switch to determine the direction of BK channel regulation by the PKA and PKC signalling cascades. Thus, activation of these important signalling pathways in β-cells may differentially regulate BK channel activity depending on the S-acylation status of the STREX insert. Intriguingly, the major S-acylating and deacylating enzymes of the STREX domain, zDHHC17 and ABHD17a, respectively, have both been linked to control of β-cell function. RNAi mediated knockdown of zDHHC17 or suppression of expression by cytokine-mediated miR146a expression in mouse β-cells [68] results in a similar phenotype to long-term BK channel loss of function including attenuated glucose-stimulated insulin secretion and apoptotic cell death [6]. Conversely, male mice with genetic deletion of ABHD17a and ABHD17c, but not ABHD17b, are reported to have improved glucose tolerance and males lacking ABHD17a also have decreased circulating insulin levels (mousephenotype.org). Thus S-acylation-dependent control of STREX variant function in β cells may be an important determinant controlling electrical excitability and insulin secretion.

Clearly understanding the role of S-acylation in controlling K_ATP_, voltage-gated Ca^2+^ channels and BK channels, as well as other ion channels that control β-cell physiology in both short and long-term control of insulin secretion and β-cell viability is warranted.

### 2.2. S-acylation of G protein-coupled receptors

A wide range of different GPCRs are expressed in pancreatic islets and activation of many of these receptors can impact insulin secretion [69]. There is a substantial literature from diverse cell types on functional effects of S-acylation on a variety of different GPCRs. As an exemplar of GPCR S-acylation relevant to insulin secretion, we focus here on recent work on the GLP-1 receptor (GLP-1R). This receptor is activated by glucagon-like peptide 1 (GLP-1), an incretin hormone released by L cells in the small intestine following a nutrient load. Activation of the GLP-1R augments glucose-stimulated insulin secretion, and GLP-1 mimetics and dipeptidyl peptidase-4 inhibitors (which block degradation of GLP-1) are used in the treatment of type 2 diabetes [70,71].

GLP-1R is internalized via endocytosis following agonist stimulation and this process is important for the effects of GLP-1 on insulin secretion. Recent work showed that the distribution of GLP-1R at the plasma membrane is regulated by S-acylation [72]. Following agonist binding, GLP-1R displayed increased clustering as visualized by both EM/immunogold labelling with nearest neighbour analysis and super-resolution microscopy in MIN6B1 β-cells [72]. Furthermore, FRET measurements between fluorescently labelled receptor and the membrane probe NR12S, which undergoes blue-shifted emission when present in liquid-ordered membranes, suggested that this clustering occurs in (cholesterol-rich) lipid-ordered domains [72]. Cholesterol depletion experiments, although lacking in selectivity, suggested that GLP-1R endocytosis was linked to its movement into these domains [72]. Importantly, agonist-induced movement of GLP-1R was shown to correlate with its increased S-acylation in MIN6B cells [72]. Furthermore, a connection between S-acylation and receptor localization was demonstrated through alanine substitution of the main S-acylation site in the receptor (cysteine-438), which led to delayed or perturbed agonist-induced receptor clustering and internalization, and a decrease in cAMP production, highlighting the functional importance of S-acylation for GLP-1R signalling [72]. Indeed, the C438A mutant supported less insulin secretion than the wild-type GLP-1R [72]. This exemplar highlights functional regulation by S-acylation of a key GPCR expressed in β-cells and how this PTM can affect receptor dynamics and micro-localization at the plasma membrane. Given the large number of GPCRs that can impact insulin signalling, the functional impact of S-acylation on this family of proteins is likely to have a diverse array of effects on insulin secretion in pancreatic β-cells [73].

### 2.3. S-acylation of the insulin granule exocytosis machinery

S-acylation also impacts proteins that are components of the insulin secretion machinery downstream of Ca^2+^ entry. Plasma membrane association of the soluble SNARE protein SNAP-25 in the HIT hamster β-cell line was blocked by substitution of a quartet of S-acylated cysteines in a central CRD [74] (figure 2). The functional importance of S-acylation was revealed in experiments comparing the ability of toxin-resistant wild-type and cysteine mutant SNAP25 to rescue Ca^2+^-stimulated secretion of human C-peptide in HIT cells treated with botulinum neurotoxin E (which cleaves endogenous SNAP25) [74]. The quadruple cysteine-to-alanine mutant of SNAP25 was only able to support approximately 10% of the C-peptide secretion supported by SNAP-25 with an intact CRD [74]. Several Golgi-localized zDHHC enzymes have been shown to modify SNAP25, with a particularly prominent role for zDHHC17, which interacts with a conserved substrate recognition motif downstream of the S-acylated cysteines in SNAP25 [75–78]. In addition to regulating SNAP25 localization at the β-cell plasma membrane, S-acylation of SNAP25 is likely to have additional regulatory effects as studies in PC12 cells suggested that the extent of S-acylation impacts membrane micro-localization, plasma membrane-endosomal cycling and exocytotic activity of this SNARE protein [29,30,79]. The potential impact of S-acylation on the association of this SNARE protein with lipid-ordered domains is intriguing as S-acylation might therefore provide a means to functionally cluster K_ATP_ , voltage-gated Ca^2+^ channels and the insulin granule fusion machinery within defined plasma membrane domains to enhance the efficiency of stimulus-secretion coupling.

The Ca^2+^ sensor synaptotagmin-7 (syt-7) is a type I membrane protein localized to insulin granules that is important for calcium-secretion coupling [26]. Syt-7 is S-acylated on three cysteine residues present in the TMD (cytosolic side) and juxtamembrane region [80]. Although it is unclear how S-acylation affects syt-7 in β-cells, work in other cell systems has highlighted the importance of these lipid modifications for intracellular sorting of syt-7. Outside of its function in β-cell exocytosis, syt-7 has been implicated in lysosomal exocytosis, a process that is important for plasma membrane repair and for the uptake of particles by phagocytosis in macrophages [81]. Experiments performed in bone marrow macrophages from syt-7 knockout mice demonstrated that cysteine-to-serine substitutions at the three S-acylation sites perturbed the ability of syt-7 to rescue phagocytosis of zymosan particles in these syt-7 null cells [82]. This loss of activity correlated with mis-localization of the S-acylation-null mutant of syt-7 in a galactosyltransferase 1-positive Golgi compartment and loss of lysosomal targeting [82]. How does S-acylation regulate sorting of syt-7 from the Golgi to lysosomes? A clue to this question came from the observation that mis-localization of the lysosomal tetraspanin CD63 (by disruption of a tyrosine-based sorting signal) also caused mis-localization of co-expressed syt-7. In this case, both the CD63 mutant and co-expressed syt-7 were routed to the plasma membrane, implying that CD63 regulates syt-7 sorting [82]. In support of this, the two proteins were shown to co-immunoprecipitate from cells and siRNA-mediated depletion of CD63 led to the accumulation of syt-7 in the Golgi [82]. Thus, S-acylation of syt-7 appears to promote either association with CD63 directly or indirectly (e.g. via scaffold proteins or recruitment into CD63 microdomains) to mediate the sorting of syt-7 to the lysosomal compartment. These analyses in bone marrow macrophages show that S-acylation of syt-7 is essential to allow it to exit the Golgi, and it is possible, therefore, that post-Golgi sorting of syt-7 in β-cells also depends on S-acylation. In this case, it will be interesting to identify β-cell proteins that sort syt-7 into insulin granules and also to understand how syt-7 evades CD63 to prevent excessive routing to lysosomes in this cell type.

### 2.4. S-acylation of proteins involved in glucose-stimulated insulin secretion highlights a broad range of regulatory effects of this post-translational modification

S-acylation exerts a variety of regulatory effects on modified proteins, many of which are exemplified in the above analysis of the glucose-stimulated insulin secretion pathway. The examples given describe the potential roles of S-acylation in promoting membrane binding, intracellular sorting, lateral segregation (and possible co-segregation) in membranes and protein–protein interactions (see summary in figure 2). The challenge now is to develop a more comprehensive understanding of how the components of this pathway are modified and regulated within their native environment and how S-acylation contributes to the overall efficiency and integration of glucose-stimulated insulin secretion within the wider β-cell physiology.

## 3. Insulin signalling and GLUT4 vesicle exocytosis

Following its release from pancreatic β-cells, insulin has wide-ranging effects on metabolism [83]. Three key targets of insulin action are hepatocytes, skeletal muscle and adipose tissue. Hepatocytes respond to increased levels of circulating insulin by repressing glucose producing pathways (gluconeogenesis and glycogen breakdown) and enhancing glycogen biosynthesis. Skeletal muscle and adipocytes increase their capacity for glucose uptake many-fold by moving the GLUT4 glucose transporter protein from an intracellular storage depot to the cell surface. Enhanced glucose uptake as a result of this redistribution of GLUT4 to the cell surface is followed by increased glycogen and triglyceride biosynthesis in skeletal muscle and adipocytes, respectively [83].

**Figure 3.**
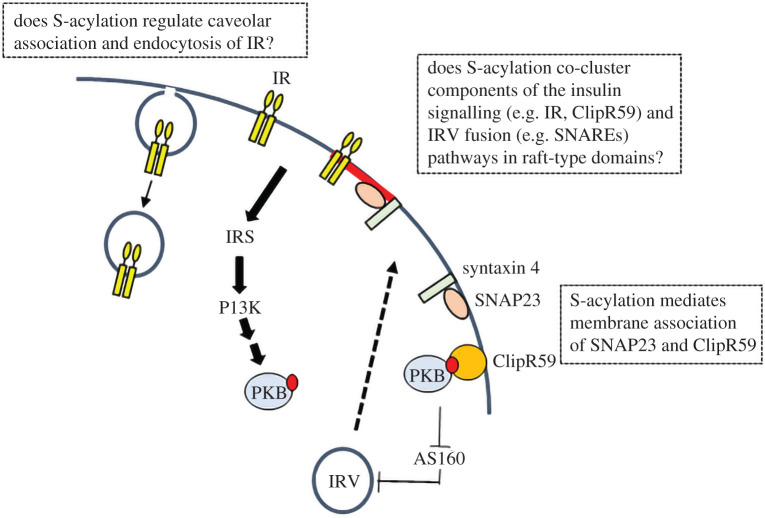
S-acylation of components of the insulin signalling and IRV exocytosis pathways. S-acylation mediates plasma membrane association of the SNARE protein SNAP23 and ClipR59, which recruits phosphorylated (and active) PKB to the plasma membrane to enhance phosphorylation of key downstream targets such as AS160, thus facilitating insulin-stimulated IRV fusion with the plasma membrane. The effects of S-acylation on the insulin receptor (IR) are not known but we speculate that it may mediate association with caveolae to regulate IR endocytosis and lipid rafts to allow the coupling to other components of the insulin-stimulated IRV exocytosis pathway such as SNAP23.

The physiological responses of hepatocytes, skeletal muscle and adipocytes to increased insulin levels are linked to the activation of an intracellular signalling cascade that involves the conversion of PIP2 to phosphatidylinositol 3,4,5-trisphosphate (PIP3) in the inner leaflet of the plasma membrane and the subsequent recruitment and activation of phosphoinositide-dependent kinase I (PDK1) [84]. PDK1 activity is required for the phosphorylation and activation of protein kinase B (PKB), which plays a central role in driving downstream effects on metabolic processes in these cells. The phosphorylation of AS160 by PKB is thought to represent a key step in the insulin-dependent translocation and SNARE-dependent fusion of insulin-responsive vesicles (IRVs) containing GLUT4 with the plasma membrane [85] (see figure 3). Our discussion will focus on the effects of S-acylation on the insulin signalling pathway leading to IRV exocytosis and on the sorting of the transporter into the specialized GLUT4 storage compartment (GSC).

### 3.1. Phosphoinositide-dependent insulin signalling pathway

Our discussion of the insulin response pathway begins with the insulin receptor, which was reported to undergo S-acylation over 30 years ago [86]. Studies in the HepG2 human hepatoma cell line demonstrated that the β subunit incorporates radiolabel via a thioester linkage in cells incubated with ^3^H palmitic acid [86]. The modification was stable, sensitive to a protein synthesis inhibitor (emetine) and was also detected on the αβ precursor of the mature insulin receptor [86]. These findings suggest that S-acylation of the receptor occurs in the early secretory pathway (endoplasmic reticulum/Golgi) during biosynthesis of the receptor [87], and that once at the plasma membrane there is no further turnover of S-acylation in HepG2 cells. The role that S-acylation plays in insulin receptor function is currently unknown; however, studies on S-acylation of the EGF receptor have identified effects of this modification on the signalling activities of the receptor, for example by switching receptor activity between PI3 K and MAPK signalling arms in the presence of oncogenic K-Ras [88]. Thus, S-acylation might also affect the signalling properties of the insulin receptor or its biosynthetic trafficking to the plasma membrane (figure 3). Another possibility is that S-acylation could also alter the microlocalization of the insulin receptor (figure 3), which could affect both the function and localization of the receptor. Indeed, there are reports that the receptor localizes to caveolae, cholesterol-rich domains at the plasma membrane, and that this is linked to endocytosis of the receptor [89]. Thus, it will be interesting to examine if S-acylation increases the preference of the insulin receptor for a lipid-ordered membrane environment and if this contributes to caveolar association [90]. As a starting point, the relative association of wild-type and S-acylation-null receptor with ordered and disordered domains in giant plasma membrane vesicles could be assessed [32].

There is no evidence that S-acylation occurs on the key components of the classical PI3-kinase-dependent signalling pathway downstream of the insulin receptor (i.e. insulin receptor substrate proteins, phosphoinositide 3-kinase, PDK1 or PKB). However, it has been shown that PKB signalling is affected by S-acylation of the regulatory protein ClipR-59 [91]. ClipR-59 was shown to interact with the kinase domain of PKB in a yeast 2-hybrid screen [91]. Further analysis of ClipR-59 uncovered an important role in recruitment of activated PKB to the plasma membrane and downstream signalling events in 3T3-L1 adipocytes. Knockdown of ClipR-59 reduced recruitment of activated (threonine-308 and serine-473 phosphorylated) PKB to the plasma membrane following insulin stimulation without affecting overall cellular PKB phosphorylation [91]. Indeed, ClipR-59 was suggested to interact selectively with phosphorylated PKB and alanine substitution of threonine-308 and serine-473 blocked ClipR-59-dependent recruitment of PKB to the plasma membrane [91]. ClipR-59 also affected the phosphorylation of PKB substrate proteins in 3T3-L1 adipocytes and, in particular, over-expression of ClipR-59 enhanced, and depletion of ClipR-59 decreased, insulin-dependent phosphorylation of AS160 and insulin-stimulated glucose transport [91]. The stimulatory effect of ClipR-59 over-expression on insulin-stimulated glucose transport was lost when the C-terminal 60 amino acids containing the S-acylated cysteine-534 and cysteine-535 residues [92] was removed, highlighting the importance of S-acylation for ClipR-59 regulation of PKB (figure 3). In the case of ClipR-59 it seems, therefore, that S-acylation is important to mediate membrane interaction of this soluble protein, allowing it to recruit activated PKB to the plasma membrane and thus directing the kinase activity of PKB towards specific substrate proteins. zDHHC17 was identified as the main enzyme responsible for S-acylation of ClipR-59, and depletion of zDHHC17 perturbed both the recruitment of activated PKB to the plasma membrane and insulin-stimulated GLUT4 plasma membrane translocation [93].

Intriguingly, zDHHC17 may also play a role in insulin signalling in adipocytes through control of STREX variant BK channels (discussed above), to regulate calcium homeostasis [94], which is important for adipocyte maturation and physiology [95]. In humans, genome-wide association studies have identified BK channels as a susceptibility locus for obesity with increased expression of mRNA encoding the pore-forming α-subunit (*KCNMA1*) in white adipose tissue and adipose tissue-derived cells [96]. Moreover, both global *Kcnma1* deletion in mice, as well as inducible conditional genetic deletion of *Kcnma1* in adult mouse adipocytes, prevents excessive body weight gain and fat deposition in response to a high-fat diet, revealing an important role for adipocyte BK channels in controlling obesity [97]. Importantly, recent studies in rats with a global deletion of BK channels reveal that loss of BK channels attenuates insulin-induced calcium influx, glucose uptake and triglyceride deposition in adipocytes [94]. Importantly, insulin-induced activation of BK channels and subsequent calcium influx in adipocytes was PI3 K-dependent, as it was prevented by the PI3 K inhibitor LY294002 but independent of elevated calcium or PKB activity. mRNA for both the ZERO and STREX variant of BK channels was expressed in mature rat adipocytes, however, in HEK293 cells only STREX variant channels were activated by insulin in a PI3 K-dependent mechanism [94]. Thus, insulin-induced activation of STREX is proposed to hyperpolarize adipocytes, that are non-excitable, and promote calcium influx most likely through members of the transient receptor family of voltage-independent calcium channels [94,97]. As STREX is S-acylated by zDHHC17 this reveals the interesting possibility that S-acylation of STREX variant BK channels controls insulin-dependent calcium influx and adipocyte physiology.

### 3.2. GLUT4 trafficking

**Figure 4.**
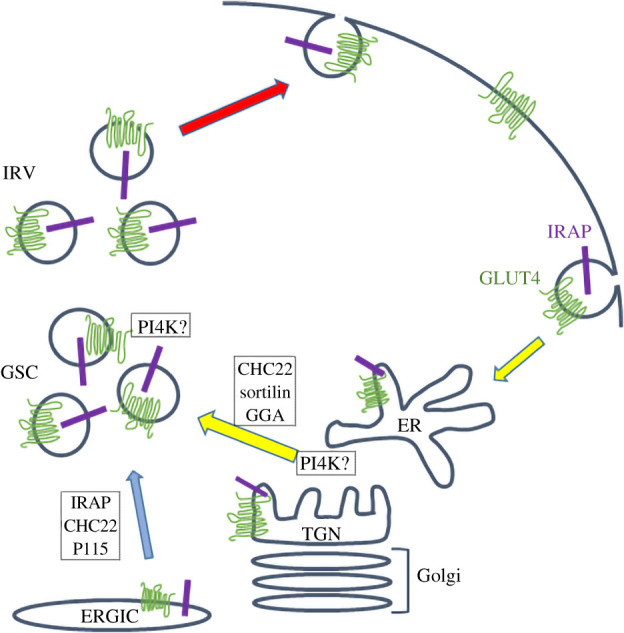
S-acylation of proteins involved in GLUT4 intracellular trafficking. GLUT4 intracellular trafficking involves multiple compartments. Newly synthesized GLUT4 can be delivered to the GLUT4 storage compartment (GSC) directly from the ER-Golgi intermediate compartment (ERGIC) and from there sorted into insulin-responsive vesicles (IRVs). Insulin stimulates the delivery of IRVs to the cell surface (red arrow), resulting in increased GLUT4 levels. GLUT4 is internalized from the cell surface into the endosomal system, and can recycle back to the GSC/IRV compartment (yellow arrows). Insulin-responsive aminopeptidase (IRAP) exhibits similar trafficking behaviour. Many of the proteins involved in key sorting steps in these inter-related cycles are S-acylated, including sortilin and PI4 K. Importantly, both GLUT4 and IRAP are S-acylated. The details of the recycling steps are minimized in this model for clarity, and the precise locus of action of some of the proteins is speculative.

GLUT4 intracellular trafficking is complex and involves multiple inter-connecting pathways including endosomes and the *trans* Golgi network and tubule-vesicular compartments throughout the cell, collectively referred to as the ‘GSC’ [98,99]. GLUT4 is further sorted into ‘IRVs’ which traffic from intracellular locations to the plasma membrane in response to insulin [98] (figure 4). Understanding GLUT4 trafficking is important as numerous studies have revealed defective GLUT4 sorting/translocation in diseases such as obesity and type 2 diabetes [100]. Below we will discuss a few examples of key regulatory proteins which are subject to S-acylation and place their role in the context of GLUT4 traffic.

The fusion of IRVs with the plasma membrane is dependent on the SNARE proteins syntaxin-4, SNAP23 and VAMP2. SNAP23 is a close homologue of SNAP25 and its membrane localization depends on the S-acylation of a cluster of five cysteine residues [75,101,102]. Similar to SNAP25, SNAP23 contains a consensus recognition site for zDHHC17, which is essential for membrane targeting, and presumably S-acylation, of this protein [75]. In addition to promoting membrane association, S-acylation also mediates the association of SNAP23 with a detergent-resistant membrane fraction following treatment of cells with Triton X-100 [30], which may indicate an underlying affinity for cholesterol-rich lipid-ordered domains at the plasma membrane of adipocytes [103]. The detergent-insolubility of SNAP23 in neuroendocrine PC12 cells was shown to be modulated by the extent of S-acylation and correlated with the ability of the protein to support exocytosis [29,30]. Thus, S-acylation may affect the function of SNAP23 in IRV fusion by increasing its partitioning into lipid-ordered domains at the plasma membrane (figure 3). It is also possible that this partitioning into specific plasma membrane domains facilitates the functional coupling of SNAP23 with other components of the signalling/trafficking pathway, including the insulin receptor.

A proteomics analysis of 3T3-L1 adipocytes showed that, in addition to SNAP23, S-acylation was also detected on other proteins linked to GLUT4 trafficking and IRV exocytosis, including Munc18c, AS160, RAB14, KIF5B, Myo1c, sortilin and PI4KIIα [101]. At this stage, the functional significance of S-acylation is unclear for most of these proteins and indeed for some the stoichiometry of S-acylation was very low. However, a few examples are discussed below as exemplars of what may turn out to be an important regulatory mechanism.

Sortilin mediates protein sorting between the Golgi and endosomal system and traffics between these compartments, with a prominent Golgi steady-state localization [104]. The role of sortilin in GLUT4 traffic appears to involve the sequestration of GLUT4 from the endosomal system into IRVs involving a complex of sortilin, CHC22 clathrin and Golgi-localized γ-ear-containing, ARF-binding protein-2 (GGA-2) which assembles on the cytoplasmic surface of intracellular vesicles (figure 4) [105,106]. Sortilin was shown to be S-acylated on cysteine-783 in HeLa cells; inhibiting acylation resulted in the accumulation of sortilin in endosomes, and expression of a sortilin-cysteine783 > serine mutant led to sortilin localizing to punctate AP3-positive endosomal structures in COS7 cells, suggesting a possible deficit in retrograde trafficking from endosomes to the Golgi [107]. The presence of the S-acylation site adjacent to the GGA-2 interacting domain of sortilin raises the intriguing possibility that this modification may regulate the formation of complexes involved in GLUT4 sorting in insulin-sensitive cells.

Trafficking of sortilin from the endosomal system to the Golgi is mediated by interactions of the sortilin C-terminus with the retromer complex [108]; a sortilin-cysteine783 > serine mutant, in contrast to wild-type sortilin, did not co-precipitate the retromer subunit VPS26 in transfected HeLa cells [107]. This has potential implications for GLUT4 trafficking, as the lumenal Vps10 domain of sortilin binds to the lumenal domain of GLUT4 and this is proposed to link GLUT4 to the retromer complex bound to the sortilin C-terminus [109]. In this way, GLUT4 is rescued from lysosomal degradation by sortilin linking GLUT4 to the retromer sorting machinery. It is not clear how S-acylation regulates the interaction of sortilin with retromer but one possibility is that S-acylation targets sortilin to a sub-domain of the endosomal system where retromer is recruited [107] and thus may regulate GLUT4 sorting.

The ability to replenish IRVs during sustained insulin stimulation is important [110,111], hence the ability to accelerate sortilin-dependent sorting of internalized GLUT4 from the endosomal system into IRVs and thus back to the plasma membrane is a key facet of GLUT4 trafficking. This involves GLUT4 traffic through multiple compartments, including endosomes and the *trans* Golgi network *en route* to IRVs (yellow arrows in figure 4) [111]. How these routes are controlled remains uncertain. Phosphoinositides and phosphoinositide kinases play a key role in the regulation of membrane traffic. Phosphatidylinositol 4-kinase Type IIα (PI4KIIα) was identified as a constituent of the GSC in both adipocytes [112] and skeletal muscle [113] where it is thought to be localized to a subset of GLUT4-positive vesicles defined by the presence of cellugyrin and the absence of sortilin [114]. These GLUT4-positive, cellugyrin-positive, sortilin-negative vesicles are thought to provide a reservoir for replenishment of the IRVs during sustained insulin action but may traffic directly to the plasma membrane in the absence of insulin [115]. How this distinction is controlled remains undefined but may involve the dynamic S-acylation of PI4KIIα. A further twist is provided by recent work showing that S-acylation and ubiquitination play opposing roles in regulating the stability and turnover of sortilin: non-S-acylated sortilin is ubiquitinated and internalized into the lysosomal compartment via the ESCRT pathway for degradation [116]. Since the sequestration of GLUT4 into IRVs is dependent upon the level of sortilin [117], this may provide a further means to fine-tune insulin action in key metabolic tissues by balancing the re-delivery of internalized GLUT4 from endosomes to the IRV.

The RabGAP protein AS160/TBC1D4 functions as a ‘brake’ on IRV exocytosis by retaining vesicles inside the cell (figure 3) [118]. Activated PKB phosphorylates AS160/TBC1D4, releasing this brake and allowing the movement of IRVs to the plasma membrane. While there remains some debate regarding which Rab isoform(s) are controlled via AS160/TBC1D4, there is considerable interest in both Rab10 and Rab14 as potential control points in GLUT4 traffic [118]. Rab10 directly facilitates IRV translocation to and docking with the plasma membrane [119]. Rab14 on the other hand mediates GLUT4 delivery to the PM via endosomal compartments containing transferrin receptor (TfR) [120,121]. It is possible that S-acylation of AS160/TBC1D4 may dictate the specificity of Rab activation. Alternatively, S-acylation of AS160 may serve to delineate distinct AS160 pools in defined cellular locations; recent work has revealed that AS160-dependent activation of Rab10 may serve to control both GLUT4 mobilization from the GSC and also the docking and fusion of IRVs with the plasma membrane [122]. How this duality of function is controlled remains presently unknown, but PTM of AS160 may underpin this behaviour.

Two other ‘hits’ identified in the S-acylated proteome analysis in 3T3-L1 adipocytes were GLUT4 and the insulin-responsive aminopeptidase (IRAP) [101], which displays tightly coordinated trafficking with GLUT4. A combination of acyl-RAC and click chemistry experiments showed that cysteine-223 is the major site in GLUT4 that undergoes S-acylation in both 3T3-L1 adipocytes and HEK293 cells [123]. This cysteine is present at the cytosol-membrane interface of TM6 of GLUT4 (the major cytosolic loop of GLUT4 is present between TM6 and TM7). The Golgi-localized zDHHC7 was identified as the main S-acyltransferase mediating S-acylation of GLUT4 [124]. When expressed in 3T3-L1 adipocytes (as HA-GLUT4-GFP), a C223S mutant of GLUT4 failed to show translocation to the plasma membrane in response to insulin stimulation and it was suggested that there was a loss of tubulovesicular localization of the mutant, suggesting a defect in sorting to the insulin-responsive compartment [123]. Indeed, confocal microscopy analysis of 3T3-L1 adipocytes suggested that expression of the GLUT4 C223S mutant also altered the localization of endogenous IRAP and inhibited translocation of this protein to the plasma membrane following insulin stimulation, and a similar effect was seen on co-expressed wild-type GLUT4 [123]. These effects suggest that the formation of the insulin-responsive IRV compartment is perturbed in cells expressing the S-acylation-null mutant of GLUT4; however, it will be important to undertake a more detailed electron microscopy analysis of GLUT4 intracellular localization and how this is affected by S-acylation.

IRAP S-acylation occurs on two cysteines at the cytosol membrane interface of this type II membrane proteins [125]. IRAP is proposed to play a role as both cargo and a regulator of the formation of the GSC/IRVs [98,126]. In part, this role is mediated by the formation of a complex between IRAP, p115 and CHC22 clathrin which is thought to control a ‘Golgi bypass’ route for GLUT4 trafficking which is particularly well-developed in human cells [105]. The molecular basis of the interactions between p115, IRAP and CHC22 remain to be resolved, but the potential that dynamic S-acylation could act to fine tune this pathway deserves attention.

Furthermore, S-acylation of GLUT4 and IRAP might contribute to the coordinated sorting and trafficking of these proteins, for example, by facilitating specific protein or lipid interactions.

An important point to consider with respect to S-acylation of GLUT4 and IRAP is the marked differences in their level of S-acylation. A semi-quantitative analysis found that the level of GLUT4 S-acylation under steady-state conditions in 3T3-L1 adipocytes was only around 12%, whereas IRAP S-acylation was determined to be approximately 60% [125]. It will be important to determine where in the cell this small fraction of S-acylated GLUT4 is localized and how the S-acylated fraction responds to insulin stimulation, compared with the non-acylated pool of the protein. An interesting approach to investigate these questions would be proximity ligation assays using clickable fatty acid probes that could facilitate selective visualization of the S-acylated pool of GLUT4 [127].

## 4. S-acylation enzymes linked to insulin secretion and insulin action

Given the emerging role of S-acylation in insulin secretion and insulin signalling, it is not surprising that several S-acylation and deacylation enzymes have been linked to these pathways and are of interest in the context of diabetes. For example, zDHHC17 emerged from a phenome–interactome analysis as a type 1 diabetes candidate protein [68]. This analysis involved combining a genome-wide linkage scan dataset with information about protein–protein interactions to prioritize candidate genes based on known interactions of the encoded protein with proteins involved in diabetes. Follow-up analysis uncovered an important role for zDHHC17 in β-cell survival and insulin secretion [68]. Although the substrate network of zDHHC17 in pancreatic β-cells has not been defined, this work clearly establishes zDHHC17 as an important S-acylation enzyme in this cell type. Established targets of zDHHC17 that might be linked to the deficits in insulin secretion following depletion of this enzyme include SNAP25, cysteine-string protein and BK channels [76,128,129]. Interestingly, the phenotypes associated with loss of zDHHC17 function (attenuated glucose-stimulated insulin secretion and apoptotic cell death) are similar to those seen with long-term BK channel loss [51].

In addition to its essential function in β-cells, zDHHC17 also appears to have a prominent role in insulin response pathways. ClipR-59, which regulates plasma membrane recruitment of activated PKB (figure 3), is also modified by zDHHC17 [93]. Over-expression of zDHHC17 in 3T3-L1 adipocytes led to increased plasma membrane levels of phosphorylated PKB, GLUT4 and IRAP under both basal and insulin-stimulated conditions compared with control cells [93]. Furthermore, plasma membrane levels of all three proteins in the presence of insulin were reduced in cells treated with zDHHC17 shRNA [93]. These effects of zDHHC17 overexpression or knockdown may be linked to loss of ClipR-59 S-acylation and/or could also reflect effects on other targets of this enzyme such as SNAP23 [75].

The zDHHC enzymes(s) mediating GLUT4 S-acylation have been investigated using co-expression experiments in HEK293T cells in which FLAG-GLUT4 was expressed with 23 mouse zDHHC enzymes. This analysis revealed that GLUT4 S-acylation was increased by zDHHC2, zDHHC3, zDHHC7 and zDHHC15 [124]. Further analysis indicated that zDHHC7 had a particularly high activity towards GLUT4 in co-expression experiments and indeed shRNA-mediated depletion of zDHHC7 (but not zDHHC3) caused a significant reduction in S-acylation of HA-GLUT4 in CHO-IR cells [124]. The investigators therefore focused on zDHHC7 and showed that depletion of this enzyme in both CHO-IR cells and 3T3-L1 adipocytes led to a reduction in S-acylation of both exogenous and endogenous GLUT4, respectively [124]. Furthermore, zDHHC7 depletion inhibited the insulin-stimulated movement of GLUT4 and IRAP to the plasma membrane in adipocytes (which is consistent with the work described above on the GLUT4 C223S mutant) [124]. Nevertheless, it is important to note that in adipocytes, knockdown of zDHHC7 also led to a 40% reduction in levels of IRAP S-acylation, and in CHO-IR cells depletion of zDHHC3 inhibited plasma membrane translocation of GLUT4 despite having minimal effect on the S-acylation of this protein [124]. Thus, some caution is required when interpreting the results of zDHHC knockdown experiments and it is important to consider effects on the wider zDHHC substrate network. However, phosphorylation of PKB was unaffected in zDHHC7-depleted 3T3-L1 adipocytes, suggesting that zDHHC7 depletion does not perturb the PI3 K insulin signalling pathway, at least [124].

Interestingly, GLUT4 S-acylation also showed a marked reduction in adipocytes from epididymal tissue, skeletal muscle and brown fat of zDHHC7 knockout mice, whereas S-acylation of GLUT4 in epididymal tissue was not affected in zDHHC3 knockout mice [124]. There was also a corresponding decrease in insulin-stimulated translocation of GLUT4, IRAP and VAMP2 to the plasma membrane in adipocytes from these zDHHC7 knockout mice [124]. The importance of zDHHC7 in the general context of insulin action was underscored by the observation that the knockout mice had increased fasting serum glucose levels and insulin tolerance tests highlighted underlying insulin resistance [124]. Furthermore, the knockout mice had elevated circulating levels of insulin. Protein S-acylation dynamics have been previously shown to be affected by insulin treatment in human umbilical vein endothelial cells (with 35 of 375 high confidence S-acylated proteins showing altered S-acylation following insulin treatment) [130], and indeed a 10-min insulin stimulation of 3T3-L1 adipocytes led to a 3-fold increase in GLUT4 S-acylation [124]. This was accompanied by a corresponding 3-fold increase in autoacylation of zDHHC7 [124]. As autoacylation is often used as a measure of zDHHC enzyme activity [2], this led to the suggestion that insulin activates zDHHC7, which then increases S-acylation of GLUT4. It is unclear how insulin treatment leads to an elevation in zDHHC7 activity but it is worth noting that zDHHC enzymes are regulated by a variety of different PTMs [131] and indeed zDHHC20 was shown to display increased tyrosine phosphorylation in insulin-stimulated 3T3-L1 adipocytes [132]. Thus, a similar phosphorylation event (or other PTM) could underlie the effects of insulin on zDHHC7 activity and GLUT4 S-acylation. To understand this model and its functional significance more completely it will be essential to gain a better understanding of the precise intracellular localization of zDHHC7—although the authors of the study suggested zDHHC7 was localized to the *trans* Golgi network [124], more recent work has suggested that this protein may be associated with the *cis* Golgi compartment [133]. It is presently unclear how dynamic changes in the activity of a *cis* Golgi enzyme might affect GLUT4 other than altering the S-acylation status of the newly synthesized protein. However, some recent observations regarding an additional route of GLUT4 trafficking are worthy of note in this context. Studies from the Brodksy laboratory have shown that a novel clathrin isoform, CHC22, expressed in humans but not (for example) rodents, plays two roles in GLUT4 trafficking [105,134]. As alluded to above, in complex with sortilin CHC22 is involved in the retrograde trafficking of GLUT4 from the endosomal system into the GSC, a pathway prominent in rodent cells. However, CHC22 also acts in concert with IRAP and p115 to sort GLUT4 directly from the endoplasmic reticulum Golgi intermediate compartment (ERGIC) into GSC/IRVs, bypassing the Golgi complex, a pathway prominent in humans [105]. Hence, alterations in zDHHC7 at the *cis* Golgi may turn out to have an effect on GLUT4 traffic by modulating the relative contributions of the two ‘routes’ taken by GLUT4 to reach the IRVs.

Although our understanding of the dynamics of protein S-acylation of the key proteins discussed in this review is limited, it is interesting to note that thioesterase enzymes that mediate deacylation of BK channels have been linked to insulin secretion and diabetes. Specifically, Lyplal1 has been associated with type 2 diabetes [57] including through the use of first-phase insulin secretion as a marker to identify candidate interacting SNPs [58], and male mice with genetic deletion of ABHD17a and ABHD17c, but not ABHD17b, have improved glucose tolerance, and males lacking ABHD17a also have decreased circulating insulin levels (mousephenotype.org).

## 5. Conclusion and future perspective

This review has highlighted key studies that reveal the emerging importance of S-acylation in insulin secretion and insulin response pathways. In particular, knockout and depletion of zDHHC enzymes clearly demonstrate the necessity of intact S-acylation pathways for efficient glucose-stimulated insulin secretion in pancreatic β-cells and for insulin regulation of GLUT4 in adipocytes. The targets of S-acylation in these cells are diverse and the effects of this modification on these proteins may be equally diverse. Our discussion has sought to highlight common aspects of S-acylation-dependent protein regulation. At one level, the effects of S-acylation are likely to be protein-specific, for example, affecting protein or membrane interactions. However, at another level, S-acylation might prove to be a central regulator of these insulin pathways by organizing pathway components to ensure their efficient interactions and maximizing efficiency within and between intracellular pathway networks, perhaps working in tandem and/or exhibiting cross-talk with phosphorylation and ubiquitination to fine-tune key processes. Indeed, the central role played by S-acylated proteins in the physiology of β-cells and insulin-responsive tissues highlights this modification as a potential new therapeutic target. Furthermore, changes in this modification, for example in response to hyperglycaemia or other homeostatic perturbations, may contribute to the development of metabolic disorders such as insulin resistance and type 2 diabetes. However, many questions remain to be answered before the true potential of targeting S-acylation as a therapeutic strategy can be realized. For example, what are the key S-acylation enzymes and substrates that are linked to insulin secretion and insulin action? How do S-acylation pathways respond to physiological and pathophysiological metabolic changes? How does S-acylation affect the function of specific proteins individually and as part of the wider signalling network? The importance of phosphorylation in insulin pathways has been studied for several decades; however, S-acylation may yet turn out to be the Cinderella of PTMs.

## Data accessibility

This article has no additional data.
